# Early Transcriptional Response to DNA Virus Infection in *Sclerotinia sclerotiorum*

**DOI:** 10.3390/v11030278

**Published:** 2019-03-19

**Authors:** Feng Ding, Jiasen Cheng, Yanping Fu, Tao Chen, Bo Li, Daohong Jiang, Jiatao Xie

**Affiliations:** 1 State Key Laboratory of Agricultural Microbiology, Huazhong Agricultural University, Wuhan 430070, Hubei, China; yfdf0310@163.com (F.D.); jiasencheng@mail.hzau.edu.cn (J.C.); boli@mail.hzau.edu.cn (B.L.); daohongjiang@mail.hzau.edu.cn (D.J.); 2 The Provincial Key Lab of Plant Pathology of Hubei Province, College of Plant Science and Technology, Huazhong Agricultural University, Wuhan 430070, Hubei, China; yanpingfu@mail.hzau.edu.cn (Y.F.); taochen@mail.hzau.edu.cn (T.C.)

**Keywords:** *Sclerotinia sclerotiorum*, Sclerotinia sclerotiorum hypovirulence associated DNA virus 1, mycovirus, transcriptome

## Abstract

We previously determined that virions of Sclerotinia sclerotiorum hypovirulence associated DNA virus 1 (SsHADV-1) could directly infect hyphae of *Sclerotinia sclerotiorum*, resulting in hypovirulence of the fungal host. However, the molecular mechanisms of SsHADV-1 virions disruption of the fungal cell wall barrier and entrance into the host cell are still unclear. To investigate the early response of *S. sclerotiorum* to SsHADV-1 infection, *S. sclerotiorum* hyphae were inoculated with purified SsHADV-1 virions. The pre- and post-infection hyphae were collected at one–three hours post-inoculation for transcriptome analysis. Further, bioinformatic analysis showed that differentially expressed genes (DEGs) regulated by SsHADV-1 infection were identified in *S. sclerotiorum*. In total, 187 genes were differentially expressed, consisting of more up-regulated (114) than down-regulated (73) genes. The identified DEGs were involved in several important pathways. Metabolic processes, biosynthesis of antibiotics, and secondary metabolites were the most affected categories in *S. sclerotiorum* upon SsHADV-1 infection. Cell structure analysis suggested that 26% of the total DEGs were related to membrane tissues. Furthermore, 10 and 27 DEGs were predicted to be located in the cell membrane and mitochondria, respectively. Gene ontology enrichment analyses of the DEGs were performed, followed by functional annotation of the genes. Interestingly, one third of the annotated functional DEGs could be involved in the Ras-small G protein signal transduction pathway. These results revealed that SsHADV-1 virions may be able to bind host membrane proteins and influence signal transduction through Ras-small G protein-coupled receptors during early infection, providing new insight towards the molecular mechanisms of virions infection in *S. sclerotiorum*.

## 1. Introduction

Mycoviruses (or fungal viruses) are prevalent in filamentous fungi and yeasts [1]. Mycovirus infections usually are associated with cryptic or latent infections, and only few known mycoviruses cause hypovirulence or hypervirulence in their host fungi [2]. The majority of research efforts, thus far, have targeted mycoviruses that are associated with hypovirulence of phytopathogenic fungi. Mycovirus-related hypovirulence has potential to develop virocontrol agents for application in fungal disease management, and to provide biological materials for research on the molecular interplay between host fungi and the invading mycovirus [3].

During long periods of co-evolution with mycoviruses, filamentous fungi have evolved antiviral defense mechanisms. However, mycoviruses have also evolved counter-defensive measures, including the activity of virus-encoded, silencing-suppressor proteins, such as p29 in Cryphonectria parasitica hypovirus 1 (CHV1). *Cryphonectria parasitica* and its associated mycovirus (CHV1) have been currently developed as a model for exploring the antiviral defense mechanism in filamentous fungi. Ten RNA silencing-related genes, including two Dicer-like (*dcl*), four argonaute-like (*agl*), and four RNA-dependent RNA polymerase genes, were identified in *C. parasitica* [4,5,6]. However, only two genes (*dcl2* and *agl2*) are required for the antiviral defense response during certain mycovirus infections. Further, it has been confirmed that a transcriptional coactivator SAGA (Spt-Ada-Gcn5 acetyltransferase) complex could regulate transcriptional induction of *dcl2* and *agl2* expression [7,8]. Recently, DCL2 has been confirmed to be bifunctional in a dual-layer antiviral defense mechanism to inhibit viral replication and alleviate symptom expression [9]. In addition, high-throughput sequencing technology provides a convenient strategy to probe the phytopathogenic fungi response to mycovirus infections at the transcriptome or proteome level, which has been applied to several filamentous fungi (*C. parasitica*, *Fusarium graminearum*, and *Colletotrichum higginsianum*, etc.) to explore potential genes or pathways responsible for symptom induction and gene alterations due to mycovirus infections [10,11,12].

*Sclerotinia sclerotiorum*, an important phytopathogenic ascomycete, has a worldwide distribution and can infect more than 600 plants, including canola, soybean, and sunflower [13]. An increasing number of published reports have shown that *S. sclerotiorum* is recognized as a suitable host for a wide variety of mycoviruses. Mycoviruses belonging to ten families, including *Hypoviridae*, *Narnaviridae*, *Alphaflexiviridae*, *Deltaflexiviridae*, *Endornaviridae*, *Partitiviridae*, *Megabirnaviridae*, *Reoviridae*, *Mymonaviridae*, and *Genomoviridae*, and two unclassified genera *Botybirnavirus* and Mycotymovirus, have been discovered and characterized in *S. sclerotiorum*, in addition to unassigned mycoviruses [14,15,16,17,18,19,20]. Thus, *S. sclerotiorum*/mycovirus provides a new interaction system for exploring the fungal response to different mycoviruses. Furthermore, previous studies have shown involvement of a serial gene in transcriptional regulation upon infection with two positive single RNA mycoviruses in *S. sclerotiorum*. Sclerotinia sclerotiorum debilitation-associated RNA virus (SsDRV) confers hypovirulence to *S. sclerotiorum*, and 150 genes with a broad spectrum of biological functions were shown to be down-regulated in response to SsDRV infection [21]. For Sclerotinia sclerotiorum hypovirus 2-L (SsHV2-L), transcriptome analysis compared virulent and non-virulent strains and found 958 mRNAs affected by the virus, of which more than 100 genes were involved in basal metabolism and transport of sugars and lipids. The authors speculated that SsHV2-L virus infection redirected the TCA cycle (tricarboxylic acid cycle ) for glycolysis and reduces apoptosis [22].

Sclerotinia sclerotiorum hypovirulence associated DNA virus 1 (SsHADV-1), a prototype virus in the family *Genomoviridae*, is the first reported fungal DNA virus. This virus is comprised of a circular single-stranded DNA genome with 2166 nt in length, and it encodes two proteins (capsid protein and replication-related protein) [23]. SsHADV-1 is related to hypovirulence of *S. sclerotiorum* and has shown great virocontrol potential under rapeseed field conditions. SsHADV-1 has several advantages as a biological agent for application in the natural environment. First, SsHADV-1 can infect a mycophagous insect, *Lycoriella ingenua*, and use it as a vector to transmit SsHADV-1 into the virus-free strain of *S. sclerotiorum* on rapeseed plants. Second, SsHADV-1 has high infectivity. This DNA virus can efficiently disrupt the vegetative incompatibility barrier of the *S. sclerotiorum* population and easily horizontally transmit among different vegetative incompatible groups. Finally, extracellular transmission is rare for mycoviruses in general. Although SsHADV-1 also has a narrow fungal host range, it can infect *S. sclerotiorum* extracellularly. The purified virions of SsHADV-1 can directly infect the hyphae of virus-free *S. sclerotiorum* on potato dextrose agar or plant leaves to effectively suppress lesion development [24,25], but the cellular receptor from fungus is still unknown for SsHADV-1.

In this study, we examined genome-wide transcriptional differences in the early stage of virions infection of *S. sclerotiorum* hyphae. It was found that cell membrane structural related proteins in the host were involved in the initial infection of virions, and some cell signal transduction processes were involved in virion invasion. This study will enhance our understanding of the response to SsHADV-1 virion infection in *S. sclerotiorum* hyphae and may provide new insights towards the molecular mechanisms of virion-induced hypovirulence of *S. sclerotiorum*.

## 2. Materials and Methods

### 2.1. Fungal Material and Growth Conditions

*S. sclerotiorum* strain Ep-1PNA367, virulent strain obtained by single-ascospore-isolation progeny of the hypovirulent strain Ep-1PN, was used [26]. Ep-1PNA367 infected with SsHADV-1 was named A367-HADV, and used for SsHADV-1 virion extraction. All strains were grown on potato dextrose agar (PDA) (Difco) plates at 20–22 °C, and stored on PDA slants at 4–6 °C.

### 2.2. Virion Extraction and Purification

The mycelia of strain A367-HADV were cultured in potato dextrose broth (PDB) (20 mL) in a stationary Erlenmeyer flask for one week at 20–22 °C. Virions were purified by sucrose density gradient centrifugation as described by Yu et al. [23]. Virions of SsHADV-1 were concentrated in 25%–30% sucrose. Subsequently, the virions were recovered by ultracentrifugation. Further, 0.01 M sodium phosphate buffer (pH 7.0) was used for virion dissolution, and virions were filtered through Millex-GP filter units (Millipore, Burlington, MA, USA). The filtered virus concentration was adjusted to 2 mg/mL, using a NanoDrop™ 2000 Spectrophotometer (Thermo Fisher Scientific, Waltham, MA, USA), and used in the infectivity assays.

### 2.3. DNA and RNA Extraction

To obtain *S. sclerotiorum* mycelium, fungal strains used in this study were cultured on PDA overlaid by cellophane membranes at 20–22 °C. DNA was extracted in CTAB following the procedures of Maniatis et al. [27]. Total RNA samples (pre- and post-inoculation with SsHADV-1 virion) were prepared using the NI-Sclerotinia sclerotiorum RNA Reagent kit (NEWBIO, Shanghai, China) according to the manufacturer’s instructions. cDNA synthesis was performed using the EasyScript® One-Step gDNA Removal and cDNA Synthesis SuperMix, according to the manufacturer’s protocol. RNA and DNA concentrations were checked and analyzed using a Nanodrop 2000 (Thermo Scientific™) machine and agarose-gel electrophoresis.

### 2.4. cDNA Library Preparation and Transcriptomic Analyses

Approximately 30 µL (3000 ng/µL) of each sample (pre- and post-inoculation with SsHADV-1 virion) were used for RNA_Seq, and RNA quality was tested using the QubitFluorometer, Agilent 2100, and NanoDrop. Total RNA was digested with DNase I and then mRNA was enriched with magnetic beads Oligo (dT). Subsequently, cDNA was synthesized using the mRNA fragments as templates. The short fragments were connected with adapters, and suitable fragments were selected for PCR amplification. Agilent 2100 Bioanalyzer and ABI StepOnePlus Real-Time PCR Systems were used in the quantification and qualification of the sample library. Following, the library was sequenced using Illumina HiSeq 4000 (BGI, Shenzhen, China, website: http://www.bgi.com/us/). After sequencing, raw reads were obtained. First, low-quality, adapter-polluted, and high content of unknown base (N) reads were screened to obtain clean reads. The clean data were deposited in the National Center for Biotechnology Information (NCBI) Sequence Read Archive (SRA) under accession: PRJNA523088. The obtained clean reads were mapped to the reference sequence of the *S. sclerotiorum* 1980 UF-70 (assembly ASM185786v1) using Bowtie2 (v2.2.5, Johns Hopkins University, Baltimore, MD, USA), and the gene expression level was calculated using RSEM (v1.2.12, University of Wisconsin-Madison, Madison, WI, USA). Subsequently, differentially expressed genes (DEGs) were detected using NOIseq, fold change ≥ 2 and probability ≥ 0.6 as screening criteria. Using the GO annotation result, DEGs were classified, and GO functional enrichment was performed using Blast2GO. KEGG annotation and enrichment were conducted using KOBAS 3.0 [28]. Subcellular localization of DEGs was predicted by the online software Deeploc-1.0. Transmembrane domains were predicted using the TMHMM Server v. 2.0 (Technical University of Denmark, Lyngby, Denmark).

### 2.5. Real-Time Quantitative Reverse Transcription PCR Analysis

RNA extraction was performed as described above. First-strand cDNA was generated from 3 μg of the same total RNA used for RNA-Seq analysis. cDNA was synthesized with an oligo d(T) primer using the reverse transcription reagent kit EasyScript® for q-PCR. Primers for the target genes were designed using Beacon Designer V7.92 and are listed in Table S6. Real-time quantitative reverse transcription PCR (RT-qPCR) was performed using q-PCR SYBR Green mix on a CFX manager system (Bio-Rad, Hercules, CA, USA) in 20 μL reactions. Each reaction consisted of 20 ng of cDNA, 0.5 μL of each primer, and 10 μL master mix. PCR reactions were performed using the thermocycler conditions: 3 min at 95 °C, 40 cycles of 15 s at 95 °C, 15 s at 57 °C, and 20 s at 72 °C. Following, melting curve analysis of amplification products was performed at the end of each PCR reaction, which verified the presence of a specific product, for 2 min at 16 °C. The ubiquitin gene served as an internal reference gene, as it is expressed stably in the nucleus [29]. In our transcriptome data, expression of the *S. sclerotiorum* ubiquitin gene (*sscle_12g088930*) did not show significant differences (Table S4, S0 as control group).

## 3. Results

### 3.1. Illumina RNA-Seq and Transcriptome Sequence Assembly

**Figure 1:**
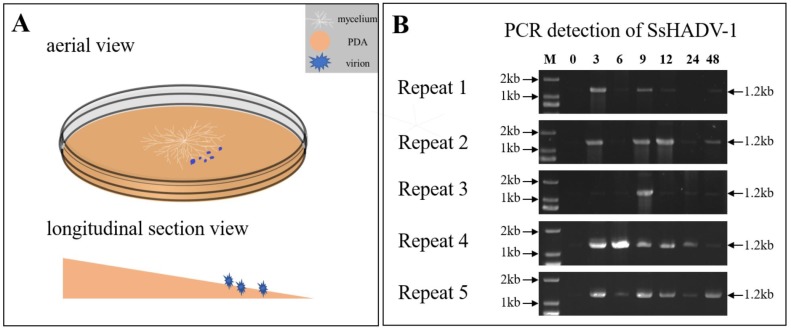
(**A**) Schematic diagram of SsHADV-1 virion inoculation on an angled plate. White, sandy brown, and blue colors represent the mycelium, PDA, and SsHADV-1 virions, respectively. After the mycelium grew on cellophane-covered PDA for 36 h, the virions were added to the lower mycelial tips. Subsequently, the upper mycelia were collected at different time points. Briefly, 15 µL of SsHADV-1 virion (2 mg/mL) were added, and strain Ep-1PNA367 was cultured under 20 °C conditions. (**B**) SsHADV-1 detection in healthy tissue after mycelia inoculation with virions at different time points (0, 3, 6, 9, 12, 24, and 48 h). SsHADV-1 was detected with specific primers (Table S6) for five biological replicates. The PCR production size was about 1.2 kb.

To identify the *S. sclerotiorum* genes involved in the response to SsHADV-1 virion infection, we analyzed the global transcriptome of *S. sclerotiorum* mycelium pre- and post-inoculated with virions of SsHADV-1 by RNA sequencing (RNA-seq). The process of SsHADV-1 virion infection of hyphae was determined before RNA-seq. The melting PDA (20 mL) was transferred into a new Petri dish, and the whole Petri dish containing PDA was placed at a small angle of inclination (about 10°) (Figure 1A). After the PDA solidified, cellophane was loaded over the PDA. Subsequently, fresh hyphae of *S. sclerotiorum* strain Ep-1PNA367 were inoculated on the center of the PDA. On the lower side of the PDA, 15 µL of purified virions (2 mg/mL) were inoculated, as the experimental treatment group S1, while the control was inoculated with virion-free buffer, as group S0 (Figure 1A). At different intervals, genomic DNA was extracted from hyphae collected on the opposite side of position inoculated virions. Following, SsHADV-1 was detected by PCR with virus-specific primers for SsHADV-1. The results showed that SsHADV-1 could be detected from mycelium at three hours post-inoculation (hpi) with virions (Figure 1B), which revealed that SsHADV-1 virions could successfully disrupt the fungal cell wall and enter *S. sclerotiorum* hyphae within 3 hpi.

Based on the above results, we collected mycelium samples at 1, 2, and 3 hpi. The samples obtained from different time points were mixed at equal amounts. The capsid gene and replication-related gene were both detected by RT-PCR in treatment group S1, but not in control group S0, suggesting that treatment group S1 had been successfully infected with virions (Figure S1). Each group contained three biological replicates. On average, 34.52 Mb Illumina raw reads were generated for each sample. After removing adapter sequences, ambiguous nucleotides, and low-quality sequences, 29.5 Mb clean reads were obtained at least for each sample. The sequencing depth exceeded 110×. The genome mapping rates for reads from virus-infected and virus-free libraries were on average 85% and 83%, respectively (Table 1).

### 3.2. Overview and Validation of Differentially Expressed Genes (DEGs)

**Figure 2:**
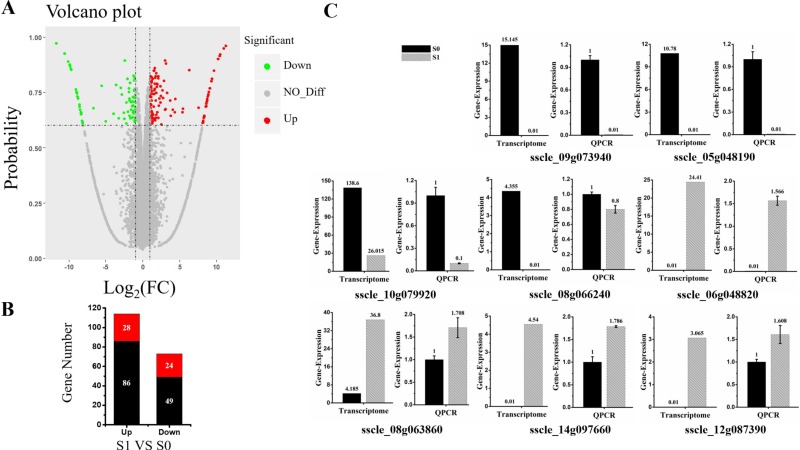
Differentially expressed genes between virus-infected and virus-free samples. (**A**) Volcano plot displaying probability on the Y-axis, index of gene differences from NOIseq, and Log2 (fold-change (FC)-value) on the X-axis. The red dots represent up-regulated differentially expressed transcripts between virus-infected and virus-free samples, green dots represent down-regulated transcripts, and gray dots indicate genes not showing statistical significance. (**B**) Stacked column showing statistics of differential genes, and red represents significantly expression genes. (**C**) The expression of eight DEGs (four up-regulated and four down-regulated DEGs) were examined via RT-qPCR. Three technical replicates were performed for each RT-qPCR. The ubiquitin gene was used as a reference gene.

Cluster analysis was carried out on the repeated samples, suggesting that S0_2 and S1_3 groups of samples could not cluster with the other two replicates in their group. In order to improve accuracy of the differentially expressed genes, the data of S0_2 and S1_3 were removed, and the enrichment analysis was carried out for two replicates for each treatment in the present study (Figure S2). Differentially expressed genes (DEGs) were identified based on a two-fold change threshold for expression relative to the virus-free sample and probability of ≥ 0.6. Further, the “volcano plot” showed distribution of the DEGs (Figure 2A). In total, 187 S. sclerotiorum genes were differentially expressed upon SsHADV-1 virion infection. Among these DEGs, 114 DEGs were up-regulated; of which, 28 were not expressed in S0 group, but expressed in S1 group. Further, 73 DEGs were down-regulated, and 24 of the down-regulated genes were completely inhibited in virion-infected samples compared to untreated samples (Figure 2B). In addition, Cp and Rep of SsHADV-1 were expressed in S1 group, but not in S0 group (Table S1). Among the 24 genes that were completely inhibited by the virus, *sscle_08g066240*, containing a conserved mob1 domain, is homologous to the *Mob1p* gene in *Saccharomyces cerevisiae*. Mob1p is a protein kinase that controls mitotic cyclin-dependent kinase to exit mitosis, cytokinesis, and G1 gene transcription [30]. Cabbage leaf curl virus (CaLCuV) belongs to *geminiviruses*. In the infection process of *Arabidopsis thaliana,* it activates genes expressed during S and G2 and inhibits genes activity during G1 and M phase of the cell cycle. Further, it can control the process of cell division to amplify their genomes [31]. We verified this gene by RT-qPCR and found that in the virus-treated group S1, gene expression was not inhibited but slightly down-regulated (Figure 2C). Among the 28 genes specifically up-regulated, the gene *sscle_06g048820* was involved in NADH: ubiquinone oxidation-reduction reactions. Combined with the GO (cellular component analysis), this gene was located in the mitochondria. It is involved in electron transport in the respiratory chain, and it can provide energy for virus replication. Of all the differentially expressed up-regulated genes, the gene *sscle_08g063860* was found to bind to the iron ion, and it is involved in pre-mRNA splicing. Gene annotation in Kyoto Encyclopedia of Genes and Genomes Pathway (KEGG) is K00101, and this pathway contributes to pre-mRNA splicing after spliceosome formation and prior to the first transesterification reaction, and is presumably involved in virus transcription [32]. The RT-qPCR results of these representative genes were consistent with the transcriptome data (Figure 2C). Taken together, invasion of virions partially control host genes in order to facilitate viral proliferation.

### 3.3. Analysis of Subcellular Localization of DEGs

**Figure 3:**
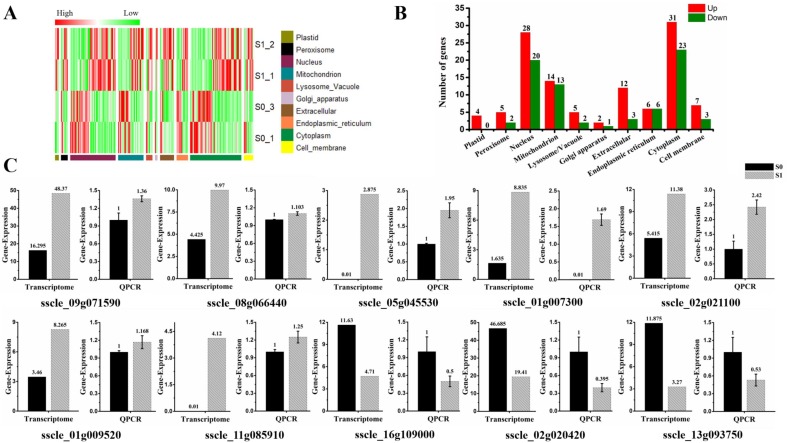
Subcellular localization of differential expression gene involved in SsHADV-1 virions infection. (**A**) Heat map enrichment analysis of all differentially expressed genes. Row clustering was carried out based on subcellular localization. The column represents the individual treatment sample. Down-regulated DEGs are displayed in green color, and up-regulated DEGs in red. The brightness of each color corresponds to the magnitude of the difference when compared against the average value. (**B**) Histogram of differentially expressed genes in various organelles, which respectively shows the number of up-regulated (red) and down-regulated (green) genes in each cell structure. (**C**) RT-qPCR validated the expression of cell membrane-related DEGs (seven up-regulated and three down-regulated DEGs). Three technical replicates were performed for each RT-qPCR. The ubiquitin gene was used as a reference gene.

The subcellular localization of 187 DEGs were predicted in different organelles. Specifically, they were distributed in 10 subcellular organelles, and most genes were predicted to be localized in the nucleus (25.7%) and cytoplasm (28.9%). Notably, four genes (*sscle_03g029040*, *sscle_12g087390*, *sscle_08g063860*, and *sscle_02g019430*) were predicted to be localized in plastids, and all of them showed up-regulated expression in this study. Depending on the features of protein solubility, 49 DEGs were related to the cell membrane, accounting for 26% of all DEGs. Of which, 10 of those membrane-related genes were predicted to be located in the cell membrane. Seven genes (*sscle_09g071590*, *sscle_08g066440*, *sscle_05g045530*, *sscle_01g007300*, *sscle_02g021100*, *sscle_01g009520*, *sscle_11g085910*) showed up-regulated expression, while three genes (*sscle_16g109000*, *sscle_02g020420*, *sscle_13g093750*) were down-regulated. The transmembrane domains of those 10 DEGs in the cell membrane were analyzed, and five genes (*sscle_01g009520*, *sscle_11g085910*, *sscle_08g066440*, *sscle_01g007300*, *sscle_05g045530*) contained transmembrane domains and were all up-regulated upon virion infection (Table S5). Further, 27 DEGs were predicted to be located in the mitochondria. Of which, 14 genes were up-regulated, and 13 genes showed down-regulated expression. The expression profiles of subcellular localization were shown with the heat map and histograms (Figure 3A,B). Cell membrane-predicted DEG expression was further verified by RT-qPCR and found that expression patterns were consistent with results of the RNA-seq analysis, with the exception of genes *sscle_08g066440* and *sscle_01g009520* (Figure 3C).

### 3.4. Small GTPase Mediated Signal Transduction in GO Enrichment

**Figure 4:**
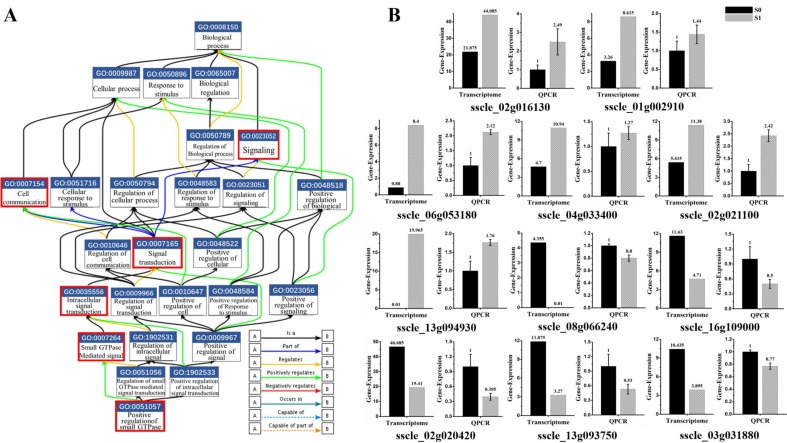
Small GTPase mediated signal transduction involved in SsHADV-1 virion infection. (**A**) Functional gene map from EMBL-EBI (The European Bioinformatics Institute). The box contains the GO number and functions, and different colored lines represent different relationships. The label in bottom-right corner is shown for details. The red box indicates the biological process by which DEGs are enriched. (**B**) DEGs were re-confirmed by RT-qPCR. Briefly, 11 DEGs (six up-regulated and five down-regulated DEGs) enriched in small G protein signaling pathways were selected. Three technical replicates were performed for each RT-qPCR. The ubiquitin gene was used as a reference gene.

The gene ontology (GO) consortium analysis is a strictly defined concept that is used widely in functional annotation and enrichment analysis in all species. GO terms were classified into three major functional ontologies (biological process, cellular component, and molecular function) [33]. A *p*-value < 0.05 was used as a threshold to select significant GO, and GO enrichment items are all listed in the supplementary figures (Figure S3A–C). GO enrichment analysis of 187 DEGs were conducted. Interestingly, about a third of annotated genes were involved in the signal transduction process mediated by Ras-small G protein. This signaling pathway consists of multiple genes to form a cascade reaction. DEG-related virion infections are mainly involved in the following molecular functional reactions: GO: 0051057, involved in positive regulation of small GTPase mediated signal transduction; GO: 0007264, related to small GTPase mediated signal transduction; GO: 0035556, responsible for intracellular signal transduction; GO: 0007165, signal transduction; and GO: 0023052, signaling (Figure 4A). Moreover, GO: 0007154 is involved in communication between cells. The expression of relevant genes involved in this pathway were further verified by real-time PCR. The results suggested that the expression pattern was consistent with that of RNA-seq analysis with exception of two genes (*sscle_08g066240* and *sscle_03g031880*). Those two genes were down-regulated more than three-fold in the RNA-seq data; however, a 0.2-fold change was measured by real-time PCR (Figure 4B). Those results revealed that signal transduction mediated by Ras-small G protein was activated upon SsHADV-1 virion infection.

In molecular function analysis, DGEs were characterized primarily in the following categories: binding and activation, including ribonucleoside and nucleoside binding (GO: 0032549 and GO: 0001882), carboxy-lyase activity (GO: 0016830), and nutrient reservoir activity (GO: 0045735). Those components are involved in basic life activities functions (Figure S3A). In terms of cell composition, it was consistent with the subcellular localization of the above genes. In addition to ribosome complex (GO: 1990904), periplasmic space (GO: 0042597), cell envelope (GO: 0030313), and outer membrane-bounded periplasmic space (GO: 0030288) were almost all enriched (Figure S3C). These results indicate that genes involved in the regulation of protein synthesis and cell membrane composition are affected by virion invasion.

### 3.5. KEGG Annotation of DEGs

**Figure 5:**
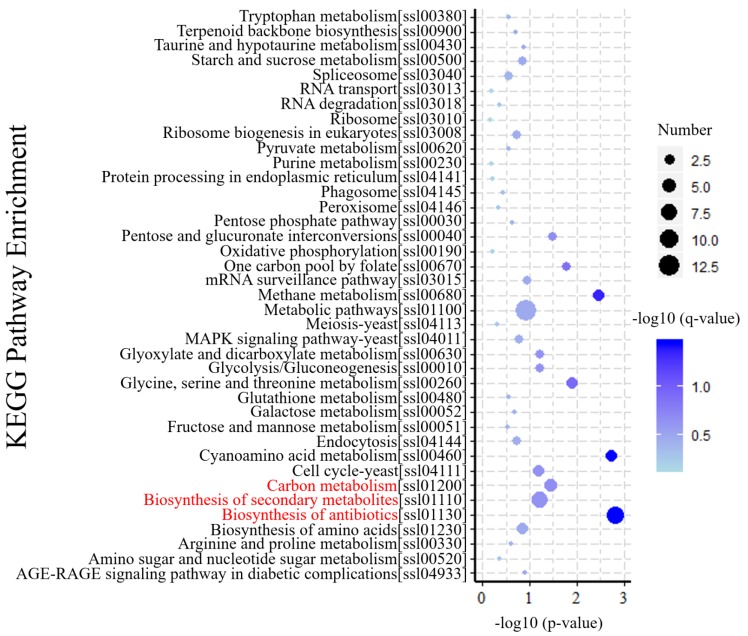
KEGG enrichment analysis of DEGs. The Y-axis of the bubble graph represents the enriched signal pathway. For each item, characters in square brackets are serial numbers related to the KEGG pathway. The X-axis is −log10 (*p*-value). The bubble size represents the number of genes, and the color is corrected p-value. The brightness of the blue color corresponds to the magnitude of reliability in the enrichment.

KEGG is a database resource for understanding the metabolic processes of biological systems from genomic and molecular information [34]. KEGG pathway analysis revealed specific pathways induced or suppressed by virus infection. To understand gene interactions with various biological functions, we used KOBAS 3.0 software for enrichment analysis of differential gene metabolism. The results of those analyses indicated that 39 KEGG pathways were mapped by 187 DEGs. As shown in (Figure 5), biosynthesis of antibiotics (ko01130) and secondary metabolites (ko01110), and carbon metabolism (ko01200) were the top three enriched pathways, which maybe play important roles in response to SsHADV-1 infection. In addition, amino acid-related metabolic pathways, including cyanoamino acid metabolism (ko00460), methane metabolism (ko00680), glycine, serine, and threonine metabolism (ko00260) were enriched. These results indicate virion invasion may disrupt the balance of amino acid metabolism in the host.

## 4. Discussion

In this study, *S. sclerotiorum* genes involved in the early response to SsHADV-1 infection were investigated. To summarize interactions in the initial stage of infection, SsHADV-1 virions first break through the fungal cell wall structure and interact with proteins related to the cell membrane. Ras-small G protein is used for signal transduction to transmit the signal of virus invasion to various subcellular structures, including the nucleus. The adjacent cells can also communicate with each other. During the above processes, the virus can modulate the differential expression of mitochondrial genes and subvert cellular biosynthetic machineries, providing energy for its own invasion and proliferation.

The time point that SsHADV-1 virions successfully enter *S. sclerotiorum* is unclear, based on our results. We could occasionally detect SsHADV-1 in the hyphae of *S. sclerotiorum* at one hour post-inoculation with virions; however, SsHADV-1 transcripts could be detected at three hours post-inoculation in all repeated experiments. Notably, SsHADV-1 accumulation was not consistent across individual treatment times in five repeated experiments, and the SsHADV-1 concentration reduction period occurred between six hours and 12 hours after inoculation with virions (Figure 1B). During this period, we speculated that an antiviral reaction was activated, inhibiting SsHADV-1 expression. Related information could be found, from the KEGG enrichment pathway, that both antibiotic and secondary metabolite biosynthesis were enriched, and related genes were mostly up-regulated (Table S2).

Mycovirus is usually regarded as lacking an infection stage in vitro; however, SsHADV-1 virions can directly infect healthy mycelia. Similarly, influenza virus can infect the human body directly. The influenza virus usually binds to hemagglutinin receptors in epithelial cells of the nose, throat, and lungs and attach to the host cell surface [35]. Subsequently, the viral envelope fuses with the cell membrane and enters the host cytoplasm in the form of vesicles through pinocytosis. The intracellular details are still being elucidated. Finally, virions enter the target endosome for genome release [36,37]. After mosquito injection of arbovirus Zika virus (ZIKV) into the skin of a mammalian host, this virus infected permissive cells via the phosphatidylserine receptor AXL and stimulated cellular autophagy by enhancing ZIKV replication in permissive cells [38]. Plant viruses cannot directly infect healthy plants, and their primary vectors for transmission are insects or infected wounds. The tobacco mosaic virus (TMV) successfully infected plant tissue within 12 h. Its replication complexes (VRCs) could interact with cortical endoplasmic reticulum, and its movement is dependent on filamentous actin and myosin in plants [39]. In combination with the invasion studies of animal viruses and plant viruses, we presume SsHADV-1 could bind to special receptor proteins on the surface of *S. sclerotiorum* cells. Membrane-related proteins involved in SsHADV-1 infection can be used as candidate proteins for experimental verification. There was no significant difference in expression of the actin gene (*sscle_14g099090*) pre- and post-inoculation with virions, but endocytosis in the KEGG metabolic pathway was enriched. Two genes (*sscle_13g094930* and *sscle_03g026360*) enriched in this metabolic pathway were significantly up-regulated, suggesting that SsHADV-1 may enter the fungal cell through endocytosis after binding to the cell membrane (Table S3).

Small G proteins regulate a wide variety of processes in the cell. Small G proteins with a molecular weight of 20–30 KDa can function independently as a hydrolase enzyme that binds and hydrolyzes guanosine triphosphate (GTP) (inactive) to form guanosine diphosphate (GDP) (active) [40]. Human astrovirus (HAstV) is a positive-strand RNA virus that can cause gastroenteritis. In the early steps of HAstV infection, Rab7-small GTPase participates in viral transport to endosomes to reach the cytoplasm and begin their replication cycle [41]. RGP1, a Ras-small GTPase in tobacco, regulates changes in SA and pathogenesis-related protein levels. Overexpression of this small GTP binding protein can interfere with normal signal pathways, affecting cytokinin biosynthesis and resulting in increased resistance to TMV infection [42]. MjCdc42 is a crucial member of the Rho-small GTPase family in kuruma shrimp (*Marsupenaeus japonicus*). White spot syndrome virus (WSSV), a dsDNA virus in the family *Nimaviridae*, employed MjCdc42-hijacking tactics to promote interactions between Marsupenaeus japonicus arginine kinase (MjAK) and WSSV envelope protein VP26, thereby enhancing WSSV replication in shrimp [43]. In our study, multiple genes regulating small G protein signal transduction were differentially expressed. However, the specific regulatory process requires further study.

In addition, four plastid-like genes were predicted to be up-regulated in organelle structures (Table S5). The plastid is a membrane-bound organelle found in the cells of plants, algae, and some other eukaryotic organisms. Plastids often contain pigments used in photosynthesis, and the types of pigments in a plastid determine the cell’s color [44]. *CASTOR* and *POLLUX*, two genes involved in the *Lotus japonicus* plastid, are key factors in controlling symbiotic fungal and bacterial growth into roots [45]. In fungi, the involvement of this plastid-like gene in viral interactions requires careful verification. For color deepening of the colony morphology and uneven pigmentation in the late stage of virus-infected *S. sclerotiorum*, further study must confirm whether this phenomenon is related to abnormal expression of plastid-like genes.

## Figures and Tables

**Table 1:** Summary of sequencing data.

Sample	Raw Reads (Mb)	Clean Reads	Clean Reads (Mb)	Clean Bases (Gb)	Clean Reads Q30(%) ^b^	Clean Reads Ratio(%) ^c^	Total Mapping Ratio ^d^	Sequencing Depth ^e^
S0_1 ^a^	35.05	30,212,592	30.21	4.53	95.28	86.19	83.80%	120ⅹ
S0_2 ^a^	33.46	29,391,054	29.39	4.41	95.27	87.84	84.39%	117ⅹ
S0_3 ^a^	35.05	30,596,914	30.60	4.59	95.65	87.29	82.51%	121ⅹ
S1_1 ^a^	35.05	30,378,866	30.38	4.56	95.57	86.66	84.01%	121ⅹ
S1_2 ^a^	35.05	30,624,970	30.62	4.59	95.94	87.37	85.72%	121ⅹ
S1_3 ^a^	33.46	29,485,096	29.49	4.42	95.78	88.12	86.05%	117ⅹ

^a^ S0_1, S0_2, S0_3: Three biological replicates of SsHADV-1 free sample. S1_1, S1_2, S1_3: three biological replicates of SsHADV-1 infection sample; ^b^ Clean Reads Q30(%): Percentages of clean bases whose correct base recognition rates are greater than 99.9% in total bases; ^c^ Clean Reads Ratio: Clean reads/raw reads; ^d^ Total Mapping Ratio: Genome mapping reads/raw reads; ^e^ Sequencing depth: Clean bases/genome size.

## Supplementary Materials

The following are available online at https://www.mdpi.com/1999-4915/11/3/278/s1, Figure S1. Two pairs of different virus primers to detect the sample’s cDNA. Figure S2. Cluster analysis between samples. Figure S3. Gene ontology analysis of DEGs. Table S1. Statistics table of virus sequencing data. Table S2 Gene expression in KEGG: Biosynthesis of antibiotics and secondary metabolites. Table S3 Gene expression in KEGG: Endocytosis. Table S4 Transcription data of internal reference genes. Table S5 Differential gene expression for subcellular localization (partial). Table S6 Oligonucleotide primers used for PCR and RT-qPCR.
