# Supplementary material for: Early Transcriptional Response to DNA Virus Infection in Sclerotinia sclerotiorum
Source: Viruses. 2019 Mar 19;11(3):278. doi: 10.3390/v11030278 (PMC6466436; doi:10.3390/v11030278)
Supplement: Supplementary file 1 [file viruses-11-00278-s001.pdf]

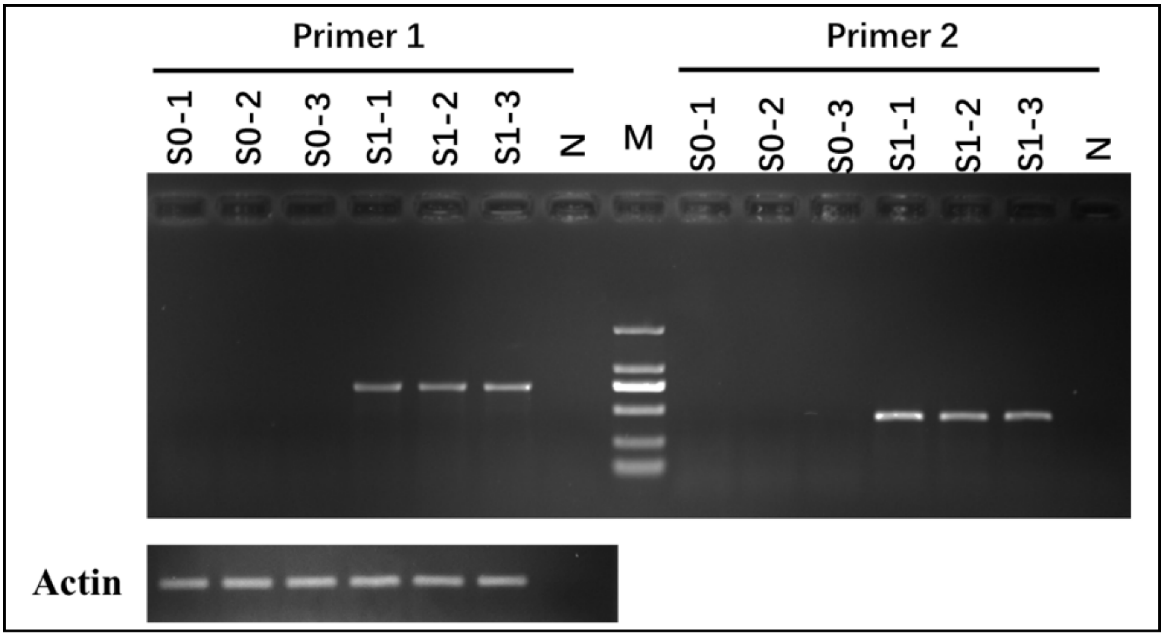

Figure S1. Two pairs of different virus primers to detect the sample's cDNA. The left Primer1 is for the virus Cp primer detection, the right Primer2 is for the virus Rep primer detection. The DNA marker is where DL2000 is. S0 and S1 represent the experimental group and the control group, respectively, biologically repeated three times. N, PCR negative control; M, DNA marker DL2000.

A B

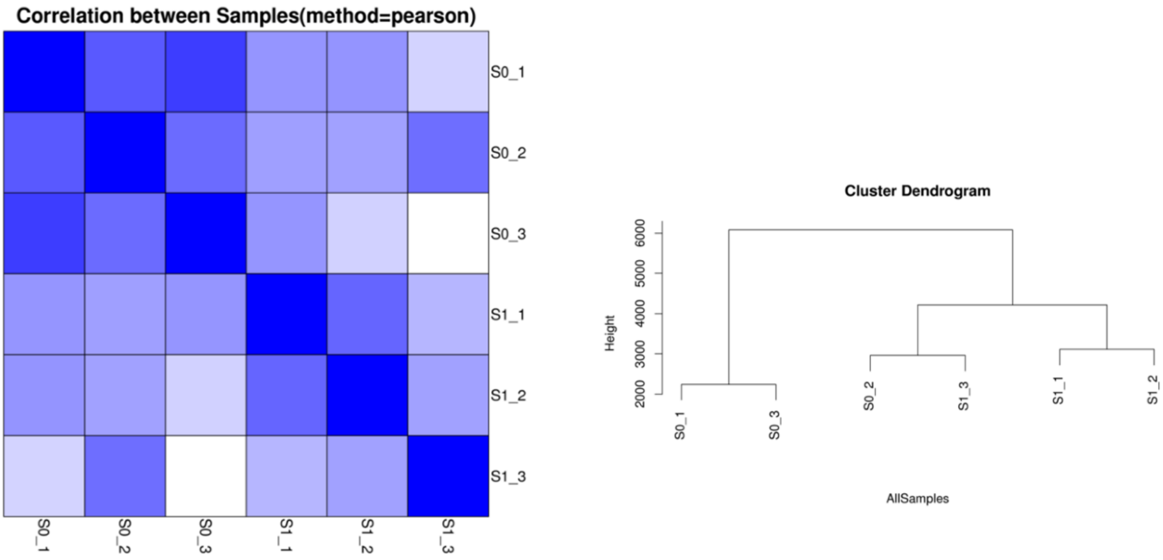

Figure S2. Cluster analysis between samples. (**A**) Heatmap of Pearson correlation between samples. Both X- and Y-axis represent each sample. Coloring indicates Pearson correlation (high: blue; low: white). (**B**) Hierarchical clustering between samples. The closer the samples the more similar the expression profile between samples.

A

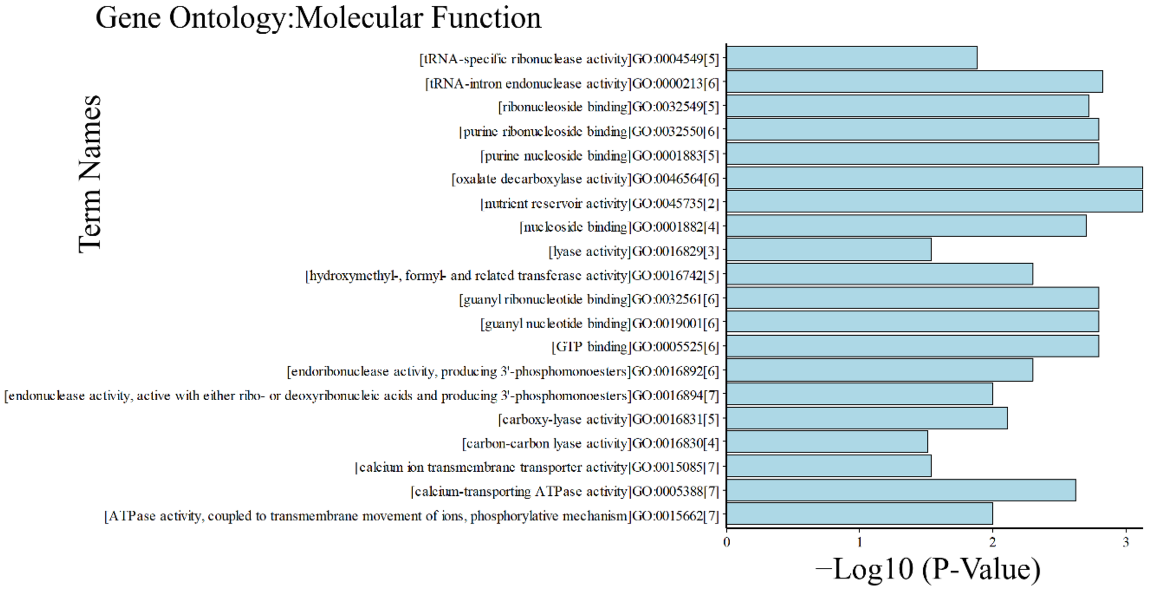
Gene Ontology:Molecular Function
Term Names
[tRNA-specific ribonuclease activity]GO:0004549[5]
[tRNA-intron endonuclease activity]GO:0000213[6]
[ribonucleoside binding]GO:0032549[5]
[purine ribonucleoside binding]GO:0032550[6]
[purine nucleoside binding]GO:0001883[5]
[oxalate decarboxylase activity]GO:0046564[6]
[nutrient reservoir activity]GO:0045735[2]
[nucleoside binding]GO:0001882[4]
[lyase activity]GO:0016829[3]
[hydroxymethyl-, formyl- and related transferase activity]GO:0016742[5]
[guanyl ribonucleotide binding]GO:0032561[6]
[guanyl nucleotide binding]GO:0019001[6]
[GTP binding]GO:0005525[6]
[endoribonuclease activity, producing 3'-phosphomonoesters]GO:0016892[6]
[endonuclease activity, active with either ribo- or deoxyribonucleic acids and producing 3'-phosphomonoesters]GO:0016894[7]
[carboxy-lyase activity]GO:0016831[5]
[carbon-carbon lyase activity]GO:0016830[4]
[calcium ion transmembrane transporter activity]GO:0015085[7]
[calcium-transporting ATPase activity]GO:0005388[7]
[ATPase activity, coupled to transmembrane movement of ions, phosphorylative mechanism]GO:0015662[7]
0
1
2
3
−Log10 (P-Value)

B

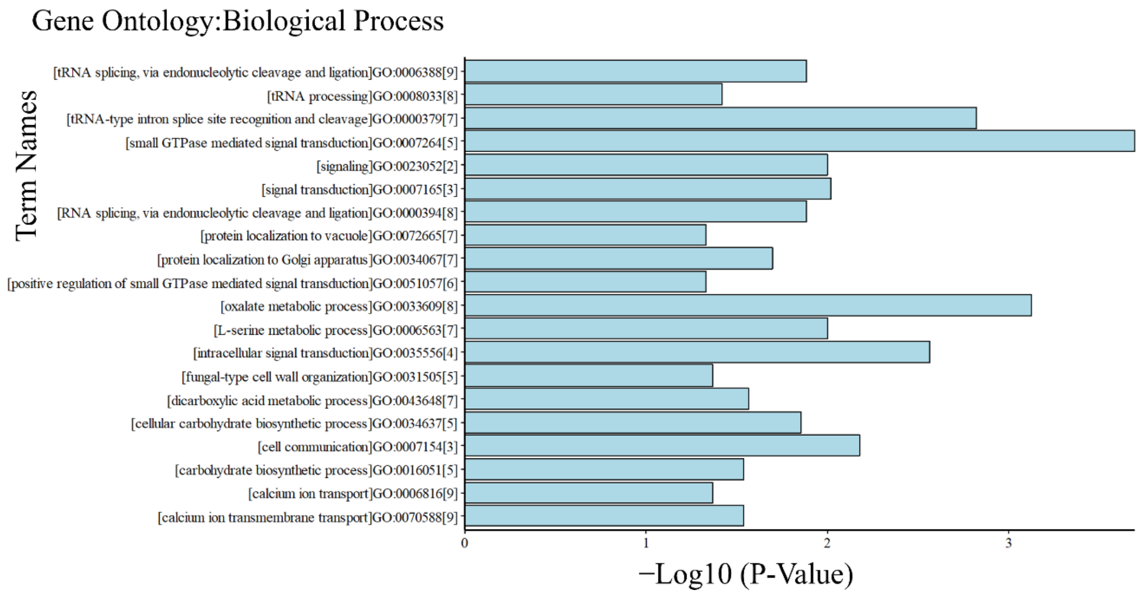
Gene Ontology:Biological Process
Term Names
[tRNA splicing, via endonucleolytic cleavage and ligation]GO:0006388[9]
[tRNA processing]GO:0008033[8]
[tRNA-type intron splice site recognition and cleavage]GO:0000379[7]
[small GTPase mediated signal transduction]GO:0007264[5]
[signaling]GO:0023052[2]
[signal transduction]GO:0007165[3]
[RNA splicing, via endonucleolytic cleavage and ligation]GO:0000394[8]
[protein localization to vacuole]GO:0072665[7]
[protein localization to Golgi apparatus]GO:0034067[7]
[positive regulation of small GTPase mediated signal transduction]GO:0051057[6]
[oxalate metabolic process]GO:0033609[8]
[L-serine metabolic process]GO:0006563[7]
[intracellular signal transduction]GO:0035556[4]
[fungal-type cell wall organization]GO:0031505[5]
[dicarboxylic acid metabolic process]GO:0043648[7]
[cellular carbohydrate biosynthetic process]GO:0034637[5]
[cell communication]GO:0007154[3]
[carbohydrate biosynthetic process]GO:0016051[5]
[calcium ion transport]GO:0006816[9]
[calcium ion transmembrane transport]GO:0070588[9]
0
1
2
3
−Log10 (P-Value)

C

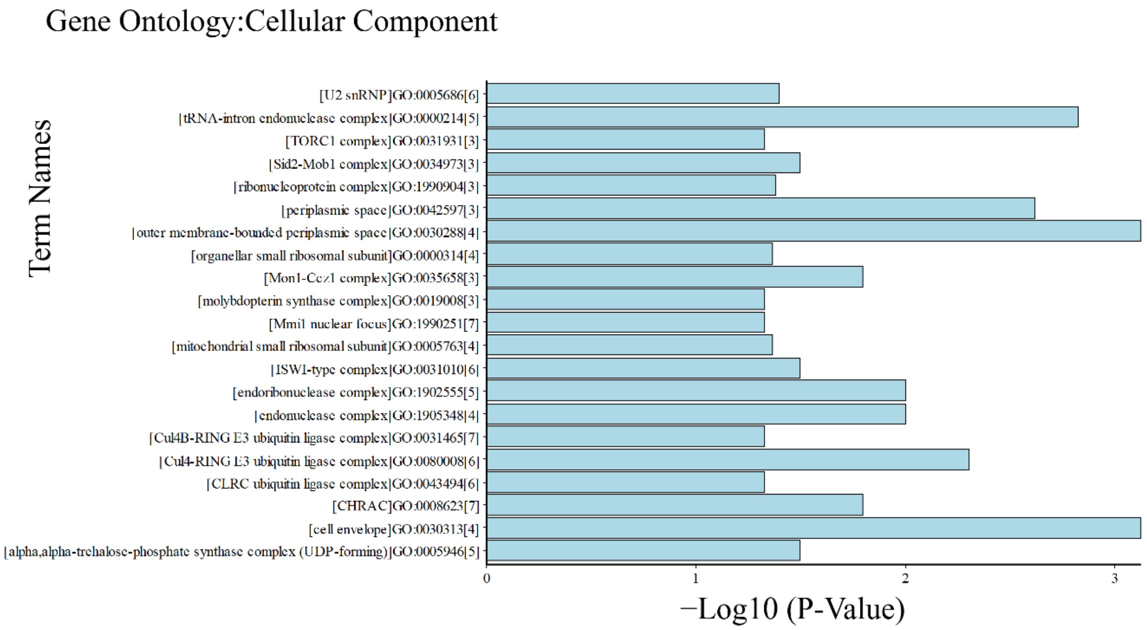

Figure S3. Gene ontology analysis of DEGs. GO has three ontologies: (**A**) Molecular function, (**B**) biological process, and (**C**) cellular component. In each bar chart, the X-axis represents −Log10 (p-value), the Y-axis represents GO term. Each number in square brackets at the Y-axis is the level of the hierarchical relationship.

Table S1. Statistics table of virus sequencing data.

	SsHADV-Cp		SsHADV-Rep	
	count[c]	FPKM[d]	count[c]	FPKM[d]
S0_1[a]	0	0	0	0
S0_2[a]	0	0	0	0
S0_3[a]	0	0	0	0
S1_1[b]	3	0.83	1	0.26
S1_2[b]	3	0.79	1	0.25
S1_3[b]	6	1.71	0	0

[a]S0_1, S0_2, S0_3: Three biological replicates of SsHADV-1 free sample.
[b]S1_1, S1_2, S1_3: Three biological replicates of SsHADV-1 infection sample.
[c]Counts are the number of reads overlapping Cp or Rep gene.
[d]FPKMs means fragments per kilobase of exon per million reads, which describes the amount of gene expression.

Table S2. Gene Expression in KEGG: Biosynthesis of antibiotics and secondary metabolites.

ID	S0-Expression	S1-Expression	log2FoldChange (S1/S0)	Probability	Up/Down (S1/S0)
sscle_02g012230	0.01	6.67	9.381543	0.812541	Up
sscle_12g087390	0.01	3.065	8.259743	0.635478	Up
sscle_12g087390	0.01	3.065	8.259743	0.635478	Up
sscle_03g029040	0.295	6.26	4.407376	0.719771	Up

sscle_12g089330	0.34	4.705	3.790588	0.645031	Up
sscle_05g045470	3.79	13.99	1.884126	0.774562	Up
sscle_08g064900	1.96	6.65	1.762501	0.636415	Up
sscle_09g069640	9.915	1.25	–2.98768	0.770863	Down
sscle_02g018280	9.555	0.01	–9.90011	0.8737	Down
sscle_09g073940	15.145	0.01	–10.5646	0.92629	Down

Table S3. Gene Expression in KEGG: Endocytosis.

Gene ID	S0-Expression	S1-Expression	log2FoldChange (S1/S0)	Probability	Up/Down (S1/S0)
sscle_13g094930	0.01	19.965	10.96326	0.947879	Up
sscle_03g026360	6.14	14.43	1.22106	0.712607	Up

Table S4, Transcription data of internal reference genes.

Gene ID	S0-Expression	S1-Expression	log2FoldChange (S1/S0)	Probability	Up/Down (S1/S0)	Gene
sscle_12g088930	6993.305	6581.405	–0.08758	0.295355	-[a]	Ubiquitin
sscle_14g099090	728.495	857.41	0.235066	0.530439	-[a]	Actin

[a]- indicates no significant difference

Table S5. Differential gene expression for subcellular localization (partial).

Gene ID	S0-Expression	S1-Expression	log2FoldChange (S1/S0)	Probability	Up/Down (S1/S0)	Localization[a]	PredHel[b]
sscle_13g093750	11.875	3.27	–1.86056	0.751194	Down	Cell membrane	0
sscle_16g109000	11.63	4.71	–1.30405	0.690831	Down	Cell membrane	0
sscle_02g020420	46.685	19.41	–1.26616	0.824108	Down	Cell membrane	0
sscle_02g021100	5.415	11.38	1.071467	0.64667	Up	Cell membrane	0
sscle_08g066440	4.425	9.97	1.171916	0.640583	Up	Cell membrane	12
sscle_01g009520	3.46	8.265	1.256243	0.618619	Up	Cell membrane	6
sscle_09g071590	16.295	48.37	1.569683	0.848131	Up	Cell membrane	0
sscle_01g007300	1.635	8.835	2.433939	0.733961	Up	Cell membrane	1
sscle_05g045530	0.01	2.875	8.167418	0.618854	Up	Cell membrane	1
sscle_11g085910	0.01	4.12	8.686501	0.708439	Up	Cell membrane	5
sscle_02g019430	4.345	19.105	2.136522	0.822375	Up	Plastid	0
sscle_08g063860	4.185	36.8	3.136406	0.8811	Up	Plastid	0
sscle_03g029040	0.295	6.26	4.407376	0.719771	Up	Plastid	0
sscle_12g087390	0.01	3.065	8.259743	0.635478	Up	Plastid	0

[a]Localization: DGEs in subcellular localization.

[b]PredHel: Number of predicted transmembrane domains.

Table S6. Oligonucleotide primers used for PCR and RT-qPCR.

Gene ID	Name	Gene primers (5'→3')	Gene ID	Name	Gene primers (5'→3')
sscle_02g016130	6130F	CGTGACTTGGTTCGTGTT	sscle_09g071590	71590F	ACTTGGATGTTGCCTTACCTAA
	6130R	TCGTAGTATTGGAGGTTCTTCT		71590R	AGATGGAATGCTGACACTTGA
sscle_01g002910	2910F	ACAGAAGTCGCATCGCTAT	sscle_08g066440	06440F	TATGGTTGGCGTGAGGTA
	2910R	ATCATCAGAAGGAGGAAGTTGT		06440R	ACTGTGGCGACTATCCTT
sscle_06g053180	3180F	AGATGGGAGTGGAAATGAAGGA	sscle_05g045530	45530F	ACGGAGGAGCATAAGGAAGAG
	3180R	TGATGTGGCGAATAGCAAGTG		45530R	GAGGCACAGCGGTATGAATG
sscle_04g033400	3400F	TGATGTTGATGATGAAGAGGAA	sscle_01g007300	07300F	TCTGCCCTTGCTTTGTCTCT
	3400R	GCCATAGTTTCTGCTGAGT		07300R	AGCCACAGCACCATCCAT
sscle_02g021100	1100F	GAGAGTGATACGGTGGTTAGATTG	sscle_02g021100	21100F	GAGAGTGATACGGTGGTTAGATTG
	1100R	GCGATAGTAGAGGCGGGATA		21100R	GCGATAGTAGAGGCGGGATA
sscle_13g094930	4930F	ATTTACCGTGTGGGATGT	sscle_01g009520	09520F	CTGTCCGCTACGCTATTCG
	4930R	ATCTCTAAGTTCGTCCTCATT		09520R	ACCGCATACCTTCCATCATTC
sscle_16g109000	9000F	GCTGAAGACAAGGATGGAAAT	sscle_11g085910	85910F	GACACCCAACAGCAAGAA
	9000R	TGGTGATTGGACTGTGACT		85910R	CTCGCCCTTCCTCATTTG
sscle_08g066240	6240F	GACTTCCTTCATCACTACC	sscle_16g109000	09000F	GCTGAAGACAAGGATGGAAAT
	6240R	CAAGCCACTCATTCTCAT		09000R	TGGTGATTGGACTGTGACT
sscle_02g020420	0420F	GAGATGATGAGCCTACGA	sscle_02g020420	20420F	GAGATGATGAGCCTACGA
	0420R	TTGTTGTTGCGGACTTAG		20420R	TTGTTGTTGCGGACTTAG
sscle_13g093750	3750F	GCTACTTCCCAACGCACTA	sscle_13g093750	93750F	GCTACTTCCCAACGCACTA
	3750R	ATAATCTTCTTGTCCCGCTGTAT		93750R	ATAATCTTCTTGTCCCGCTGTAT
sscle_03g031880	1880F	CGCCTTACCAATCGCTAA	sscle_08g063860	63860F	CCTTCTCCTCCCTTTCCAT
	1880R	ACACTCTTCAAATCCAATCTCT		63860R	TGATGTTGATGTAATGCGATGA
sscle_09g073940	73940F	GCATTCCTGTTGGCTGATA	sscle_14g097660	97660F	TAAGGAGTGCGAGGAAGAA
	73940R	TGGTAGTGGTGACGACAT		97660R	TATCCAGCATCAGCAGTAGA
sscle_05g048190	48190F	GGACAAGGAGGACAAGAG	sscle_12g087390	87390F	CGGTTGAGACGGCGATTA
	48190R	CCACCACCGTATGAGTTAT		87390R	TTGCGGTCATAGTTCTTCCAT
sscle_10g079920	79920F	AACGGCAACAGCAGAATC	sscle_12g088930	ubiquitin-F	CCACCTCACCTACATCTTC
	79920R	ACGAACATCAGTCCCTCAA		ubiquitin-R	CGTAGCATCGGATAATGTTG
sscle_06g048820	48820F	TGGTTACGGTCTCAATGG	SsHADV-1	SsHADVLF	ATTTTGTTTAAGGGGGCAGAGAATG
	48820R	ATGCTCCTTCTTGTGATGTA		SsHADVLR	CGGACGGAAGTTTCGCAGTAGA
